# Maturing Out of Problematic Alcohol Use

**Published:** 2004

**Authors:** Patrick M. O’Malley

**Affiliations:** Patrick M. O’Malley, Ph.D., is a research professor at the University of Michigan’s Institute for Social Research and co-principal investigator on the Monitoring the Future study, both positions in Ann Arbor, Michigan

Problematic alcohol use—that is, heavy drinking, or drinking that is accompanied by unpleasant consequences—tends to increase as people go through late adolescence, peaking at about age 22 or so, and then decline as they grow older. Some researchers consider this decline, which has been studied for more than 70 years in many different countries and cultures (Fillmore 1988; Johnstone et al. 1996), a “maturing out” of problem drinking. This process is believed to result when people reach their twenties and take on the roles and responsibilities of adulthood (Bachman et al. 2002; Yamaguchi and Kandel 1985). (For information about alcohol use among young people in other cultures, see the article in this issue by Ahlström and Österberg.)

The transition to adult roles typically involves ending formal education and beginning employment; becoming financially independent of parents; establishing new living arrangements (alone, cohabiting, married); and getting married and starting a family. All of these events are associated with declines in problematic alcohol use. For some young people, the first transition to adulthood, however—leaving the parental home, whether to attend college or not—often coincides with an increase in heavy alcohol use (O’Malley and Johnston 2002). This sidebar first examines how leaving the parental home is associated with increases in heavy alcohol consumption, and then looks at two transitions of young adulthood that are strongly associated with declining heavy alcohol use among young adults: marriage and parenthood.

## Transitions to Independence

### Leaving Home

A variety of models have been proposed to explain how this first transition period supports or encourages problematic drinking (Schulenberg and Maggs 2002). (See the article by Schulenberg and Maggs in this issue.) Often (though by no means always), the first transition is entrance into a college environment, which seems to be particularly likely to be accompanied by heavy drinking. Researchers have found it difficult to determine whether people begin drinking more heavily in college in response to the social environment (that is, it is a socialization effect) or as a function of their own enduring personality characteristics (that is, it is a selection effect; for example, when young people who are more likely to drink are more likely to go to college) (Yamaguchi and Kandel 1985; Labouvie 1996).

Both selection and socialization effects may be operating here to varying degrees, but there is little doubt that the social environment of college exerts a strong influence on student drinking. Much problematic alcohol use among college students occurs in group settings (Wechsler et al. 1995). Fraternity parties are notorious for encouraging excessive drinking, but student gatherings in bars or other settings often entail heavy drinking as well. (See the article by Saltz in this issue.) A longitudinal study by McCabe and colleagues (2005) has provided strong evidence for both selection and socialization effects among U.S. college students who join fraternities and sororities: Higher drinking rates were seen before they attended college, and membership in a fraternity or sorority was associated with considerably greater than average increases in heavy drinking.

Because the social environment of college generally supports alcohol use, it is reasonable to expect that leaving college could result in declines in heavy-drinking rates (Sher et al. 2001). And, in fact, the end of formal higher education—and concomitant transitions to employment and independent living—does signal a period of maturing out of one role into a role that normally involves more responsibility and less freedom.

Young people who do not attend college tend to show drinking patterns that resemble those of college students, with heavy alcohol use increasing until about age 22—particularly among those who leave the parental home—and then declining as these young people take on adult responsibilities (Bachman et al. 1997). More research on alcohol use among 18- to -22-year-olds not attending college is needed to elucidate the factors that influence drinking behavior among this under-studied group.

### Getting Married

Perhaps the major transition affecting alcohol use is marriage, which is well known to be associated with a decline in problematic drinking (Leonard and Rothbard 1999). Early studies examining the relationship between marriage and problem drinking at a single point in time (i.e., cross-sectional studies) have shown that married young adults may have lower problematic use of alcohol than those who are single, and this difference may reflect selection effects. That is, young people who drink less may be more likely to marry, or to marry earlier, than those who drink more. Longitudinal studies, which track a group of people over time, provide the best evidence on why alcohol use declines after marriage; these studies indicate that marriage itself helps reduce alcohol consumption, particularly heavy alcohol use (Bachman et al. 1997; Bachman et al. 2002; Leonard and Rothbard 1999).

What is it about marriage that produces these reductions in alcohol use? Probably the major contributing factor is that marriage ushers in a change in social and recreational activities. Married people have fewer evenings out for fun and recreation, attend fewer parties and other social events, get together less often with friends, and do not go to bars, taverns, and nightclubs as much. Getting engaged to be married also tends to take people out of the singles scene, which helps reduce heavy drinking, although becoming engaged does not appear to have as strong an effect on social life (and on heavy drinking) as marriage does (Bachman et al. 2002; Leonard and Mudar 2003).

### Having Children

Another major transition period that almost certainly accounts for a decrease in heavy alcohol use is pregnancy. Women may reduce their alcohol use during pregnancy for many reasons (Coles 1994). Young women generally believe that alcohol can be toxic to a developing fetus; some find that the physiological changes of pregnancy render the taste of alcohol unpleasant. Having a pregnant spouse does not appear to affect men’s drinking behavior, however (Bachman et al. 1997).

Parenthood seems to impact social life even more than marriage does. Child care responsibilities limit the amount of time people have for going out with a spouse or with others. Becoming a parent, like getting married, seems to mean fewer get-togethers with friends, fewer parties and other social events, and fewer visits to bars, taverns, and nightclubs. Single mothers experience similar restrictions on socializing.

Becoming a parent appears to be the key event that prompts men, especially men who have been heavy drinkers, to reduce their drinking (Bachman et al. 1997).

## Changing Attitudes

Several researchers have suggested that young people mature out of heavy alcohol use because, along with adult responsibilities, they also adopt more conventional attitudes and outlooks (Donovan et al. 1983). Considerable evidence indicates that people’s attitudes and beliefs about alcohol are strongly associated with their drinking behavior. Investigators have questioned whether changes in attitudes or beliefs lead to changes in drinking patterns. One longitudinal study concluded that when people become engaged, marry, become pregnant, and have children, they tend to become more disapproving of heavy drinking and aware of its risks, and this change in attitude may help explain their reduced alcohol use (Bachman et al. 2002). On the other hand, the same study showed that when they reach college, young adults tend to approve of drinking more and perceive it to be less risky than they did before college, which could explain their increased alcohol use.

## Summary

When many young people have their first taste of independence from parental restrictions, but before they take on the responsibilities of adult life, they tend to increase their alcohol use. College students probably drink more because the social environment of college encourages excessive drinking. Their age-mates who do not attend college but live apart from their parents also increase their rates of heavy drinking. After about age 22, young people begin to assume more adult responsibilities—employment, independent living, and especially marriage and parenthood—that are associated with less heavy alcohol use. This maturing out can be attributed partly to the limitations these events place on social activities in general and possibly also to changes in these young adults’ attitudes toward drinking. For particularly problematic alcohol users—those who would be diagnosed as alcohol dependent—predisposing personality characteristics may mean that they are not as drawn to the more stabilizing choices of marriage and parenthood, or that these milestones do not affect their drinking behavior (Matzger et al. 2004). That is, for alcohol-dependent young adults, selection effects may be stronger than socialization effects in determining drinking behavior.
